# Supplementary material for: The genome sequence of the Gelatinous Scale Worm, Alentia gelatinosa (Sars, 1835)
Source: Wellcome Open Res. 2023 Nov 22;8:542. [Version 1] doi: 10.12688/wellcomeopenres.20176.1 (PMC10980856; doi:10.12688/wellcomeopenres.20176.1)
Supplement: Peer review history

# Reviewer report: Reviewer response for version 1

**DOI:** 10.21956/wellcomeopenres.22339.r77382

**Published:** 2024-03-29

**Authors:** Gaspar Jekely

**Affiliations:** 1 Centre for Organismal Studies (COS), Heidelberg University, Heidelberg, Germany; 2 Living Systems Institute, University of Exeter, Exeter, UK

This paper presents the genome sequence and chromosome-scale assembly of the annelid worm Alentia gelatinosa from a single specimen. The authors combined long-read DNA sequencing, Hi-C mapping and RNA sequencing to generate a high-quality resource for this species. The project is part of the Darwin Tree of Life Project and follows the protocols and best practices of the project.

 The data are presented clearly and raw data, assemblies and the analyses are available in open databases. The interested reader is encouraged to look at the very well presented interactive figures that make the analysis of the raw data accessible.

 An annotation of the genome will be presented through the Ensembl pipeline. The authors may consider posting an update or a comment linked to this paper once the annotation becomes available.

Are sufficient details of methods and materials provided to allow replication by others?

Yes

Is the rationale for creating the dataset(s) clearly described?

Yes

Are the datasets clearly presented in a useable and accessible format?

Yes

Are the protocols appropriate and is the work technically sound?

Yes

Reviewer Expertise:

annelid molecular biology, transcriptomics, neurobiology

I confirm that I have read this submission and believe that I have an appropriate level of expertise to confirm that it is of an acceptable scientific standard.

# Reviewer report: Reviewer response for version 1

**DOI:** 10.21956/wellcomeopenres.22339.r75028

**Published:** 2024-03-05

**Authors:** Stéphane Hourdez

**Affiliations:** 1 Observatoire Océanologique de Banyuls, Sorbonne Université, Banyuls-sur-Mer, France

This data paper reports the sequencing and assembly of the Polynoidae *Alentia gelatinosa* (Annelida, Polychaeta). The family Polynoidae is the most speciose (~1000 described species worldwide) of the scale worm families (Aphroditiformia). This genus is mono-specific and belongs to the subfamily Lepidonotinae, which also comprises *Lepidonotus clava*, already sequenced by the DToL initiative. This adds to the growing resources for the scale worm families. The methods used are proven and reliable. The resulting assembly is high quality and spans 1.24 Gbases, scaffolded into 15 chromosomal pseudomolecules (16-18 in other species).

 This will be a very valuable resource for the scientific community worldwide. In particular, 6 Polynoidae genomes are now available (4 shallow-water, 2 deep-sea hydrothermal vent), which allows comparative genomics studies. No major problem was found. A minor point is that with 4 sallow-water species (2 Lepidontinae, 1 Acholoinae and 1 Polynoinae), comparative studies could be carried out. In particular, genome size differences (ranging from 1 to 1.5 Gbases) could reflect partial genome duplications, or vast variations in transposable elements copies. He et al. 2023 have shown some important chromosomal translocations between Harmothoe impair and Branchipolynoe longqiensis. Can we find others compared to Alentia gelatinosa.

Are sufficient details of methods and materials provided to allow replication by others?

Yes

Is the rationale for creating the dataset(s) clearly described?

Yes

Are the datasets clearly presented in a useable and accessible format?

Yes

Are the protocols appropriate and is the work technically sound?

Yes

Reviewer Expertise:

Phylogeny and evolution of adaptations in invertebrates. Taxonomy of annelids, especially Polynoidae.

I confirm that I have read this submission and believe that I have an appropriate level of expertise to confirm that it is of an acceptable scientific standard.

## References

[1] : Genomic Analysis of a Scale Worm Provides Insights into Its Adaptation to Deep-Sea Hydrothermal Vents. *Genome Biol Evol* .2023;15(7) : 10.1093/gbe/evad125 10.1093/gbe/evad125 37401460 PMC10348049
